# Knowledge and Awareness of Varicose Veins and Its Risk Factors in Al-Qunfudah, Saudi Arabia

**DOI:** 10.7759/cureus.60266

**Published:** 2024-05-14

**Authors:** Medhat Taha, Yahya Soliman Alhilaly, Mohammed Adel alghamdi, Abdulrahman Ali Alharbi, Abdulrhman Abdullah Alhazmi, Mazen Mohammed Alzelaei, Mohammed Ali Alquzi, Faisal Ali Alhasani, Ibrahim Omar Alquzi

**Affiliations:** 1 Department of Anatomy, Umm Al-Qura University, Al-Qunfudhah, SAU; 2 College of Medicine and Surgery, Umm Al-Qura University, Al-Qunfudhah, SAU; 3 Department of Medicine, General Hospital of Al-Qunfudhah, Al-Qunfudhah, SAU

**Keywords:** saudi arabia, risk factors, prevalence, population, attitude, awareness, knowledge, varicose vein

## Abstract

Background

Varicose veins are defined as visibly swollen and twisted veins, surrounded sometimes by patches of flooded capillaries. Varicose veins are a relatively common condition. For many people, they are a family trait. Women are at least twice as likely as men to develop them.

Aim

This study aims to assess the public knowledge and awareness of varicose veins in Al-Qunfudah, Saudi Arabia.

Methods

A correlational cross-sectional study was conducted among a sample of people in Al-Qunfudah, Saudi Arabia. An online questionnaire was used for data collection. The data collection sheet included socioeconomic-demographic information. Varicose vein knowledge was assessed in the second portion using three-point ratings. The final section had multiple-choice questions about risk factors and complications, including symptoms, diagnostic techniques, and risk factors for varicose veins.

Results

Participants were included in the study after excluding individuals aged less than 18 years old, with a majority being males (319; 79.9%). Regarding age distribution, participants aged 36-45 years constituted the largest group (132; 33.1%). Out of 399 participants, 369 (92.5%) had not been diagnosed with varicose veins. Most participants (271, 67.9%) had heard of varicose veins, with the primary sources of information being someone they knew with varicose veins (106, 39.11%). Family history was considered an important factor by 141 (35.3%) of respondents while 217 (54.4%) were unsure. Female gender and old age showed significantly higher knowledge levels (p< 0.05).

Conclusion

The current study concluded that while there was a fair level of public awareness of varicose veins in general, there was a noticeable lack of knowledge regarding clinical symptoms and diagnostic techniques. Younger females demonstrated noticeably greater awareness and comprehension of the illness.

## Introduction

The deep fascia anatomically divides the lower limb venous system into two distinct circulatory systems: the subfascial or deep venous system and the epifascial or superficial venous system [1]. The deep veins drain the muscles and bones and are typically associated with their arteries. The superficial veins, on the other hand, drain the skin and muscles subcutaneously. The two main superficial veins, the great saphenous vein and the little saphenous vein, eventually empty into the deep veins either directly or by perforating veins [2].

Asia has a higher prevalence of varicose veins (VV) than the West, with Saudi Arabia having a 62% incidence. Varicose veins are found in 10% to 60% of people globally [3]. Insufficient venous valves can result in venous congestion and high venous pressure, which causes VV to be abnormally enlarged, convoluted, and visible. Because of saphenous vein insufficiency, they are more common in the lower extremities [4]. The initial signs and symptoms include weariness, heaviness in the legs, and nighttime cramps. Paresthesia, a dull or burning pain, and swelling in the dorsum of the foot and medial and lateral ankles are what comes next. Notable anomalies in the lower limbs include spider veins or wiggly-shaped little swellings beneath the skin's surface. The location of the limb affects the size of the soft, painless swellings [5].

Advanced age, obesity, pregnancy, constipation, smoking, a positive family history of the condition, a history of venous thrombosis, a sedentary lifestyle, high blood pressure from standing for extended periods of time, and heavy lifting are risk factors for VV [4,6,7]. Even though people in obesity class III reported severe limb symptoms, which are a marker of chronic venous insufficiency, obesity is not thought to be a risk factor in and of itself [8]. Sometimes, people confuse VV for a cosmetic problem. On the other hand, they may result in serious side effects like pain, discomfort, leg cramps, ulceration, a reduced quality of life, etc. Maintaining a healthy weight, avoiding standing for extended periods of time, regular exercise, eating a high-fiber, low-salt diet, keeping the legs up when sitting or lying down, avoiding wearing high heels or tight socks or stockings, and switching up your sitting and standing positions throughout the day are some preventive measures to take to avoid VV [9]. Few studies have been conducted to learn more about the causes and issues related to VV in Al-Qunfudah. Therefore, the current study intends to bridge this research gap by evaluating the knowledge and awareness of the Al-Qunfudah population regarding VV.

## Materials and methods

A descriptive cross-sectional study was conducted targeting the entire population in Al-Qunfudah, Saudi Arabia, during the period from January to March 2024. The selection of participants was predicated upon the subsequent inclusion criteria: Male and female participants from the Saudi Arabian Al-Qunfudah population between the ages of 18 and 65 years. Anyone below the age of 18 years, those who refused to give written informed consent for participation, and adults who had progressive unexplained weight loss, bloody diarrhea, or recurrent fever were excluded from the study. Data were collected using Google Forms, an Arabic-language online self-administered questionnaire that was constructed by the study researchers, and the link was shared on social networking sites, including Telegram, WhatsApp, and X (previously Twitter). Authors specifically targeted accounts and social groups that already contained Al-Qunfudah locals, and we also included a request that the questionnaire be distributed among family and friends via WhatsApp groups. We also included a statement asking recipients to disregard the message if they had already completed the questionnaire in social group messaging to prevent duplicate responses. There were three sections on the questionnaire. The sociodemographic traits of age, sex, marital status, income, educational attainment, nationality, employment status, height, and weight were the main topics of the first segment. Varicose vein knowledge was assessed in the second portion using three-point ratings (Yes, No, I don't know), which included information on prior VV diagnoses. Do you know what VV is called? Is VV known by any other names? Do you consider it to be a serious illness? Do distinct varieties of VV exist? Does a person's family history provide a risk? Does it impact those under 40? Do you know of any lifestyle modifications that can lower risk factors? Do you engage in volunteer efforts to raise awareness of VV? The final section had multiple-choice questions about risk factors and complications, including symptoms, diagnostic techniques, and risk factors for VV.

Sample size and sampling technique

Determination of the sample size was done by using the Raosoft calculator (http://www.raosoft.com/samplesize.html). The study site was specifically chosen to be the province of Al-Qunfudah Governorate in Makkah. The population of Al-Qunfudah is around 300,516 according to the most recent report from 2022. A total of 377 was the bare minimum sample size that was anticipated when a 95% confidence level and 5% margin of error were used. The participants were selected randomly at the time of collection. Situated in the Tihamah plain on Saudi Arabia's Red Sea coast is the governorate of Al-Qunfudah. With a western-facing view of the Red Sea, it is the fourth most populous province in the Makkah Region. Both Makkah and Jeddah are about 350 and 360 kilometers south of it, respectively. 19.1281° North, 41.0787° East is the position on the map. The sample units were recruited through an electronic survey that was distributed via various electronic platforms, and it was used to gather data for these three months. Ethical approval was obtained from the Biomedical Research Ethics Committee of Umm Al-Qura University, Al-Qunfudhah, Saudi Arabia (approval number: HAPO-02-K-012-2024-02-2017).

Statistical analysis

The statistical analysis was done by SPSS (version 26, IBM Corp., Armonk, NY, US). The categorical data (including awareness and knowledge about varicose veins) were presented as frequencies and percentages. In addition, the chi-square test was used to compare the sociodemographic data and awareness of VV. The results of the chi-square test showed frequencies, percentages, and p-values. Predictors of awareness regarding VV were assessed using a multivariable binary logistic regression analysis, where the significantly associated variables in the bivariate analysis were entered into the regression model as independent variables and the awareness level was employed as a dependent variable. Results were expressed as odds ratios (ORs) and 95% confidence intervals (95% CIs). A p-value of < 0.05 is an indication of statistical significance.

## Results

Sociodemographic data

The study assessed the knowledge and awareness levels among the Al-Qunfudah population regarding VV and its risk factors. A total of 399 participants were included in the study after excluding individuals aged less than 18 years old, with a majority being males (319; 79.90%). Regarding age distribution, participants aged 36-45 years constituted the largest group (132; 33.10%). In terms of educational background, the majority had a university degree (294; 73.70%), while in terms of occupation, most participants were employed (272; 68.20%). The vast majority of participants were Saudi nationals (391; 98.00%). When considering physical characteristics, most participants fell within the height range of 150-169 cm (240; 60.20%) and weight range of 70-109 kg (218; 54.60%) (Table 1).

**Table 1:** Sociodemographic data (n = 399)

Parameter	Category	N	%
Gender	Male	319	79.90%
	Female	80	20.10%
Age	18-25	96	24.10%
	26-35	54	13.50%
	36-45	132	33.10%
	46-55	102	25.60%
	> 55	15	3.80%
Educational level	Primary	4	1.00%
	Middle school	5	1.30%
	High school	60	15.00%
	University	294	73.70%
	Masters	21	5.30%
	PhD	8	2.00%
	Other	7	1.80%
Occupation	Student	72	18.00%
	Employed	272	68.20%
	Unemployed	44	11.00%
	Retired	11	2.80%
Nationality	Saudi	391	98.00%
	Non-Saudi	8	2.00%
Height	< 150	11	2.80%
	150-169	240	60.20%
	170-190	146	36.60%
	> 190	2	0.50%
Weight	< 50	19	4.80%
	50-69	136	34.10%
	70-109	218	54.60%
	110-140	21	5.30%
	> 140	5	1.30%

Awareness and knowledge of VV

The study investigated the awareness and perceptions of VV among respondents in Al-Qunfudah. Out of 399 participants, 369 (92.50%) had not been diagnosed with VV. Most participants (271; 67.90%) had heard of VV, with the primary sources of information being someone they knew with VV (106; 39.11%). A significant portion of respondents (331; 83.00%) did not know any other names for VV. Regarding seriousness, 124 (31.10%) believed VV to be a serious condition, while 198 (49.60%) were unsure. Similarly, 152 (38.10%) were aware that there are different types of VV while 235 (58.90%) were unsure. Family history was considered an important factor by 141 (35.30%) of respondents while 217 (54.40%) were unsure. A total of 247 (61.90%) did not know if VV affects people under the age of 40 or not. Awareness about preventive measures or lifestyle changes was moderate, with 131 (32.80%) being familiar with them and 206 (51.60%) being unsure. However, most participants (383; 96.00%) had not participated in campaigns or awareness events related to VV. Regarding seeking medical care, 146 (36.60%) indicated a very high likelihood (Table 2).

**Table 2:** Awareness and knowledge of varicose veins (n = 399)

Parameter	Category	N	%
Have you ever been diagnosed with varicose veins?	No	369	92.50%
	Yes	30	7.50%
Have you heard the term varicose veins before?	No	128	32.10%
	Yes	271	67.90%
If your answer is yes to the previous question, from what source did you hear about it?	Media	84	31.00%
	Healthcare professionals	35	12.91%
	Educational curriculum	38	14.02%
	Someone you know has varicose veins	106	39.11%
	Other	8	2.96%
Are there any other names for varicose veins?	No	25	6.30%
	Yes	43	10.80%
	I don't know	331	83.00%
Do you think varicose veins are a serious condition?	No	77	19.30%
	Yes	124	31.10%
	I don't know	198	49.60%
Do you think there are different types of varicose veins?	No	12	3.00%
	Yes	152	38.10%
	I don't know	235	58.90%
Do you believe that a family history of varicose veins is an important factor in developing the condition?	No	41	10.30%
	Yes	141	35.30%
	I don't know	217	54.40%
Does varicose veins affect people under the age of 40?	No	24	6.00%
	Yes	128	32.10%
	I don't know	247	61.90%
Are you familiar with any preventive measures or lifestyle changes that can help reduce the risk of developing varicose veins?	No	62	15.50%
	Yes	131	32.80%
	I don't know	206	51.60%
Have you ever participated in any campaigns or awareness events related to varicose veins?	No	383	96.00%
	Yes	16	4.00%
On a scale of 1 to 5, how likely are you to seek medical care if you experience symptoms related to varicose veins, where 1 is very low and 5 is very high?	Very low	60	15.00%
	Low	45	11.30%
	Intermediate	94	23.60%
	High	54	13.50%
	Very high	146	36.60%

**Figure 1:**
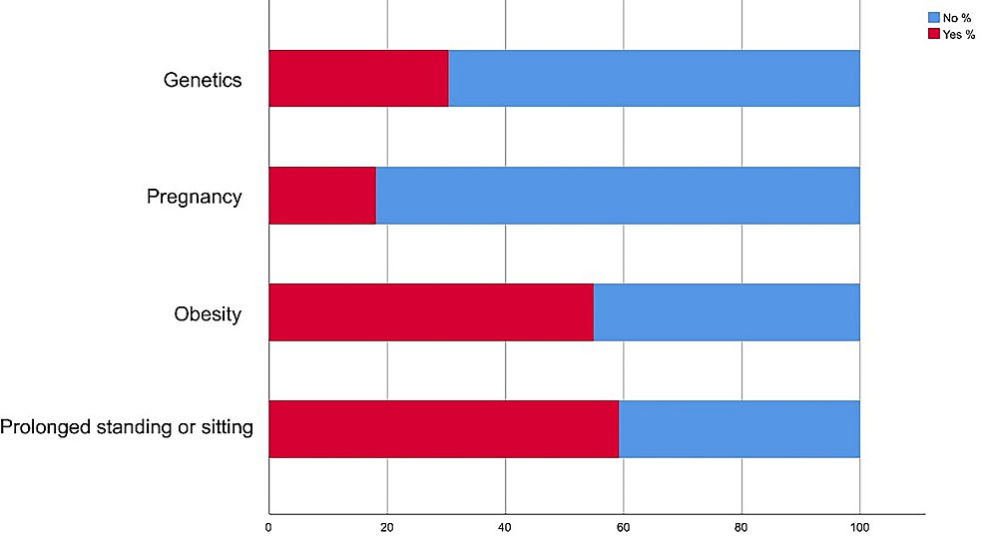
Stack bar chart showing the risk factors of varicose veins

In terms of risk factors of VV, most of the participants documented that prolonged standing or sitting had the largest percentage (236; 59.10%) (Figure 1).

**Figure 2:**
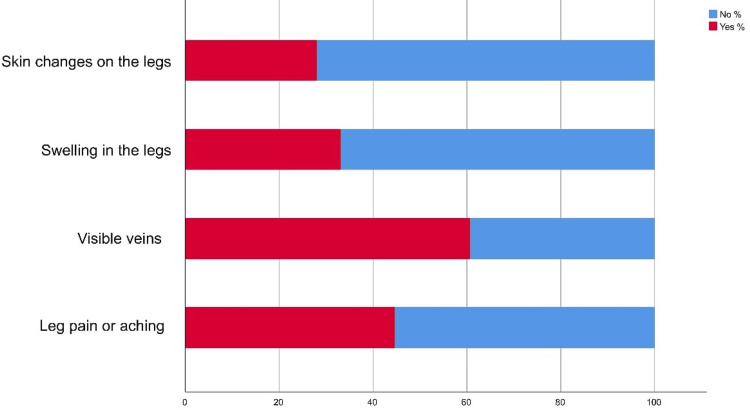
Stack bar chart showing the symptoms of varicose veins

In addition, the majority of participants reported that visible veins were the most common symptom (242; 60.70%) of varicose veins (Figure 2).

**Figure 3:**
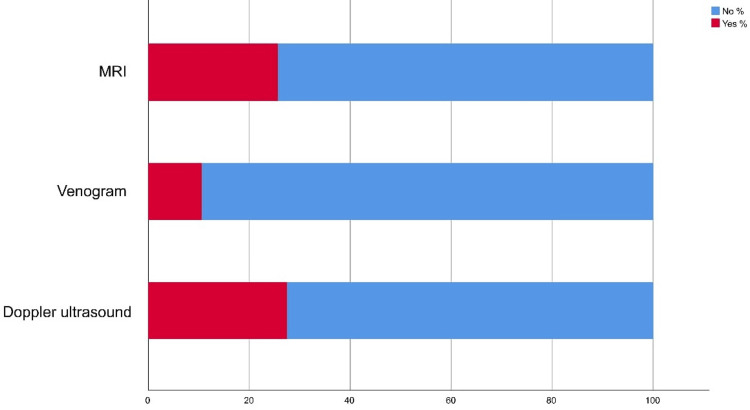
Stack bar chart showing the diagnostic methods of varicose veins

Regarding the diagnostic methods of VV (Figure 3), a large group of participants reported that Doppler ultrasound was the major method (110; 27.50%).

The association between awareness of VV and socio-demographic data

The analysis of factors influencing VV awareness among respondents in Al-Qunfudah revealed several significant associations. Gender showed a strong association, with 201 (63.00%) of males reporting an awareness compared to 70 (87.50%) of females (p-value < 0.001). Age was also a significant factor, as older participants (>55 years) were less likely to have awareness about VV 6 (40.00%) compared to younger age groups, and the age group (36-45) had high awareness (94; 71.20%) (p-value = 0.038). Interestingly, educational level, occupation, nationality, height, and weight did not show statistically significant differences in VV awareness rates among participants (p-value> 0.05 for all) (Table 3).

**Table 3:** The association between the awareness of VV and socio-demographic data

Parameter	Category	No		Yes		p-value
		N	%	N	%	
Gender	Male	118	37.00%	201	63.00%	< 0.001
	Female	10	12.50%	70	87.50%	
Age	18-25	38	39.60%	58	60.40%	0.038
	26-35	16	29.60%	38	70.40%	
	36-45	38	28.80%	94	71.20%	
	46-55	27	26.50%	75	73.50%	
	> 55	9	60.00%	6	40.00%	
Educational level	Primary	1	25.00%	3	75.00%	0.399
	Middle school	2	40.00%	3	60.00%	
	High school	24	40.00%	36	60.00%	
	University	91	31.00%	203	69.00%	
	Master's	5	23.80%	16	76.20%	
	PhD	1	12.50%	7	87.50%	
	Other	4	57.10%	3	42.90%	
Occupation	Student	26	36.10%	46	63.90%	0.575
	Employed	82	30.10%	190	69.90%	
	Unemployed	17	38.60%	27	61.40%	
	Retired	3	27.30%	8	72.70%	
Nationality	Saudi	125	32.00%	266	68.00%	0.740
	Non-Saudi	3	37.50%	5	62.50%	
Height	< 150	2	18.20%	9	81.80%	0.401
	150-169	72	30.00%	168	70.00%	
	170-190	53	36.30%	93	63.70%	
	> 190	1	50.00%	1	50.00%	
Weight	< 50	5	26.30%	14	73.70%	0.665
	50-69	40	29.40%	96	70.60%	
	70-109	73	33.50%	145	66.50%	
	110-140	9	42.90%	12	57.10%	
	> 140	1	20.00%	4	80.00%	

The odds ratios (OR) with corresponding 95% confidence intervals (CI) and p-values were calculated to assess the association between demographic factors and VV awareness among respondents in Al-Qunfudah. For gender, females showed significantly higher odds of being aware of VV compared to males, with an OR of 4.044 (95% CI: 1.983-8.247, p-value < 0.001). Among different age groups, individuals aged 46-55 years had a higher odds ratio of being aware of VV with OR of 1.851 (95% CI: 1.005-3.406, p = 0.048) compared to the reference group (18-25 years) (Table 4).

**Table 4:** The predictors of awareness of varicose veins based on the statistically significant sociodemographic data

Parameter	Category	OR	95% CI		p-value
			LB	UB	
Gender	Male	Ref.	Ref.	Ref.	Ref.
	Female	4.044	1.983	8.247	< 0.001
Age	18-25	Ref.	Ref.	Ref.	Ref.
	26-35	1.222	0.583	2.564	0.596
	36-45	1.447	0.819	2.558	0.203
	46-55	1.851	1.005	3.406	0.048
	> 55	0.464	0.150	1.433	0.182

## Discussion

The current study aimed to assess the knowledge and awareness levels among the Al-Qunfudah population toward VV and its risk factors. The findings of our work revealed that most of the study respondents were males with high education and employed. This made them more liable for risk factors of VV, including long-standing, high daily activities, and others, which may be reflected in their awareness level. The study revealed that less than 10% of the study participants were diagnosed with VV. Globally, the prevalence of VV varies from 10% to 60% [10,11]. Compared to the Western world, where it can reach 30%, the incidence is higher in the Asian region [12]. Research has indicated that the frequency in Saudi Arabia may reach up to 62%, with a yearly increase in incidence of roughly 5% for females and 2% for males [13], and this is similar to the current study's reported prevalence.

Regarding awareness of VV, the study showed that about two-thirds of the study participants head and know about VV mainly from other cases they know about or through social media. Also, a considerable portion knows about the disease's nature, age of disease onset, and preventive measures irrespective of that most of them have not participated in any campaigns or awareness events related to VV. Regarding risk factors, the most known included prolonged standing and setting and obesity. Visible veins, leg pain, swelling, and skin changes were the most known symptoms. As for diagnostic methods, study participants showed inappropriate knowledge and awareness levels where Doppler US was known for only one-fourth of them. In Hail, Khatoon et al. found that disease pathophysiology and risk factors are well known among 46% of the study group and less known by 56.0% of the study population only [14]. Also, George et al. found that the majority of respondents (70%) had a moderate level of knowledge which is similar to the current study findings [15]. Many studies assessed good knowledge levels about VV and its management [16-18]. A much lower awareness was reported by Gaire and Pathak [19] where only 6.7% of respondents heard about VV. In Saudi Arabia, no previous studies assess public awareness about VV but among other community categories. Paulsamy et al. found that 74% of the nurses had an adequate knowledge level about VV and its prevention [20].

The current study also showed that young females showed the highest awareness level of VV. This may be due to that the disease prevalence is much higher among females than males due to pregnancy and body weight variations [21,22]. Other studies failed to find any association between participants' demographic data and their knowledge of VV [23].

Study limitations

This study possesses some limitations. First, sampling bias, as we intended to reach a representative sample from the Al-Qunfudhah governorate population via a social media questionnaire, so the people who don’t have a social media account and available Internet were excluded. Second, the study being cross-sectional may affect the result's precision and generalizability, mainly it was conducted in one area in Saudi Arabia. Lastly, the accuracy of the results regarding the awareness of VV may be facing some changes in the interpretation due to variations in the educational level of participants.

## Conclusions

In conclusion, the current study showed that public awareness about varicose veins in general was satisfactory, but poor awareness about clinical symptoms and diagnostic methods was observed. Females of a young age showed significantly higher knowledge and awareness about the disease. Also, the study showed that the prevalence of VV (diagnosed cases) was within reported national and international levels. Social media and experience in other cases were the main sources of participants' information. Public awareness about VV, risk factors, and preventive measures should be improved mainly among the at-risk group. This can be achieved through periodic health education campaigns and more effort paid by health care staff. Also, motivation to practice the preventive skills of varicose veins to lead a quality life is recommended.
